# Rhytidome- and cork-type barks of holm oak, cork oak and their hybrids highlight processes leading to cork formation

**DOI:** 10.1186/s12870-024-05192-4

**Published:** 2024-06-03

**Authors:** Iker Armendariz, Unai López de Heredia, Marçal Soler, Adrià Puigdemont, Maria Mercè Ruiz, Patricia Jové, Álvaro Soto, Olga Serra, Mercè Figueras

**Affiliations:** 1 Laboratori del suro, Departament de Biologia, Facultat de Ciències, Universitat de Girona, Carrer Maria Aurèlia Campmany 40, Girona, 17003 Spain; 2 Departamento de Sistemas y Recursos Naturales. ETSI Montes, Forestal y del Medio Natural, Universidad Politécnica de Madrid, José Antonio Novais 10, Madrid, 28040 Spain; 3 Institut Català del Suro. Carrer Miquel Vincke i Meyer 13, Palafrugell, 17200 Spain

**Keywords:** Cork, Cork oak, Holm oak, Hybrids, Outer bark, Periderm, Phellem, Rhytidome, Suberin

## Abstract

**Background:**

The periderm is basic for land plants due to its protective role during radial growth, which is achieved by the polymers deposited in the cell walls. In most trees, like holm oak, the first periderm is frequently replaced by subsequent internal periderms yielding a heterogeneous outer bark made of a mixture of periderms and phloem tissues, known as rhytidome. Exceptionally, cork oak forms a persistent or long-lived periderm which results in a homogeneous outer bark of thick phellem cell layers known as cork. Cork oak and holm oak distribution ranges overlap to a great extent, and they often share stands, where they can hybridize and produce offspring showing a rhytidome-type bark.

**Results:**

Here we use the outer bark of cork oak, holm oak, and their natural hybrids to analyse the chemical composition, the anatomy and the transcriptome, and further understand the mechanisms underlying periderm development. We also include a unique natural hybrid individual corresponding to a backcross with cork oak that, interestingly, shows a cork-type bark. The inclusion of hybrid samples showing rhytidome-type and cork-type barks is valuable to approach cork and rhytidome development, allowing an accurate identification of candidate genes and processes. The present study underscores that abiotic stress and cell death are enhanced in rhytidome-type barks whereas lipid metabolism and cell cycle are enriched in cork-type barks. Development-related DEGs showing the highest expression, highlight cell division, cell expansion, and cell differentiation as key processes leading to cork or rhytidome-type barks.

**Conclusion:**

Transcriptome results, in agreement with anatomical and chemical analyses, show that rhytidome and cork-type barks are active in periderm development, and suberin and lignin deposition. Development and cell wall-related DEGs suggest that cell division and expansion are upregulated in cork-type barks whereas cell differentiation is enhanced in rhytidome-type barks.

**Supplementary Information:**

The online version contains supplementary material available at 10.1186/s12870-024-05192-4.

## Background

The periderm arises during the radial thickening of stems and roots (secondary growth) and confers protection against water loss and pathogen entrance and overall contributes to the plant fitness [1]. This protective function is afforded by the phellem, which accumulates a lignin-like polymer and a suberin polyester in their cell walls. The periderm is important in herbaceous, tubers, and some fruits but specially in woody plants, where it is the prevalent protective tissue. In woody species, a new secondary phloem is produced outwardly and a new secondary xylem inwardly from the vascular cambium every year during the growing season [2]. This newest xylem and phloem pushes the outer layers centrifugally and, generally, a new phellogen is formed within the area of the older phloem, protecting the young phloem from outside [3]. Like vascular cambium, phellogen or cork cambium is a bifacial and lateral meristem activated seasonally. Periclinal divisions of phellogen cells produce phellem outwardly and phelloderm inwardly. The structure formed by phellem (cork), phellogen, and phelloderm constitute the periderm [4]. In most woody species, and contrarily to vascular cambium, phellogen has limited activity, and successive phellogens develop in inner positions in the bark. When a new periderm is formed inward, the outer tissues including the older periderm will eventually die [3]. The newest phellogen marks the limit of the inner bark (comprising the living phloem) and the outer bark, the later usually forming a so-called rhytidome [5]. This rhytidome therefore includes successive thin, suberized and intricate phellem layers, enclosing heterogeneous cortical tissues (parenchyma, fibres, etc.) and collapsed phloem cells [4].

Noteworthy, the phellogen is thought to be active throughout the tree life in cork oak (*Quercus suber*) [6], and as such, it forms a persistent or long-lived periderm [1]. Therefore, there is a unique, thick, and continuous periderm mostly consisting of phellem cells known as cork. Cork has economic and environmental relevance. It is an industrially profitable renewable raw material and suberin chemical recalcitrance elicits CO_2_ sequestration, which is favoured by the periodic extraction of cork that stimulates the cork production between 250 and 400% [7]. Despite the uniqueness of cork oak in maintaining a persistent periderm, the cellular and molecular mechanisms that trigger its persistence by encompassing the internal growth are still largely unknown. Previous transcriptomic studies of outer barks of cork oak and rhytidome-developing oaks (*Q. ilex* and *Q. cerris*) highlighted some processes and genes enriched in rhytidome and cork but the identification of differentially expressed genes was limited due to the low-coverage offered by Roche-454 Life Sciences platform and by the lack of biological replicates [8, 9].

Cork oak shares habitat and hybridises naturally with holm oak (*Quercus ilex*) [10], a species showing the typical rhytidome. *Q. ilex* x *Q. suber* offspring differ in their outer bark anatomy, although generally they show a rhytidome-like outer bark, similar to *Q. ilex* but with significantly thicker phellem layers [11]. Our aim in this study is to identify the molecular mechanisms underlying the formation of the two main bark types, rhytidome and cork as a “single thick phellem”. For this purpose, we have included in our transcriptomic analysis not only *Q. ilex* (rhytidome) and *Q. suber* (cork) samples, but also hybrid individuals, with different introgression levels and intermediate barks. Using the Illumina platform and the cork oak draft genome [12], the comparison of cork-type and rhytidome-type barks transcriptomes provides new candidate genes of cork formation related to development, cell division, growth and differentiation. Overall, this research provides insight into the molecular basis underlying the development of different types of bark, a key protective feature of woody plants and, in the case of cork oak, with a relevant economic interest.

## Results

### Anatomical and chemical analyses to classify the outer barks of *Q. ilex x Q. suber *hybrids and the parental species

**Fig. 1:**
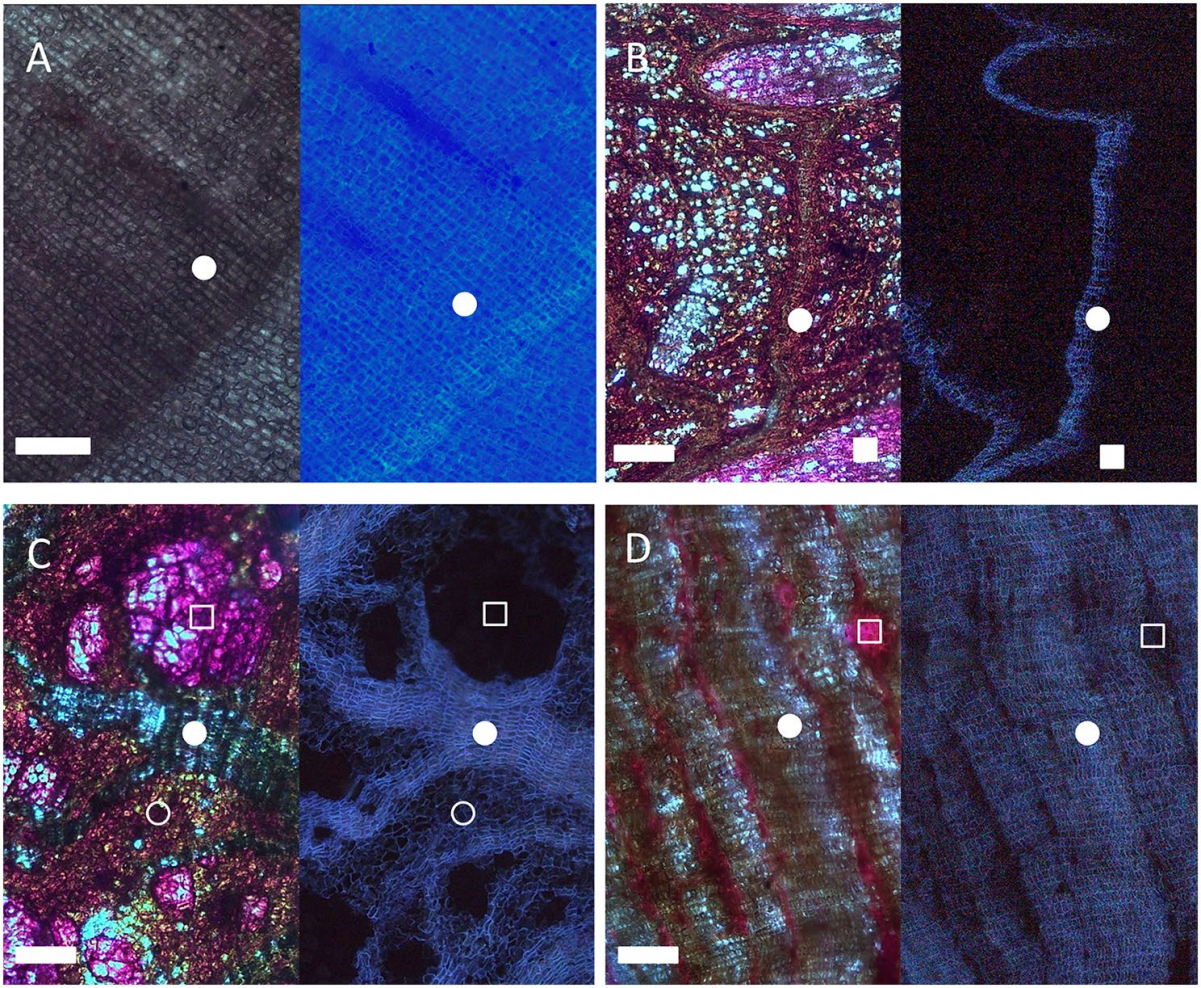
Outer bark anatomy of cork oak, holm oak and their hybrids. Suberized cell wall fluorescence detected in cross-sections under UV light after phloroglucinol-HCl staining. (**A**) Cork oak (*Q. suber*), (**B**) holm oak (*Q. ilex*), (**C**) F1 hybrid with rhytidome-like phenotype, (**D**) specific hybrid backcrossed with *Q. suber* and with a cork-type phenotype. Bright field (left) and UV (right) observations in each panel. Phellem layers (closed circle), suberized inactive phloem (open circle) and a lignified phloematic ray (closed square). Scale bars: 200 μm

Microscopic observations of cross-sections under UV light after phloroglucinol staining highlighted the suberized cell walls of the different outer barks used in this study (Fig. 1). The outer bark of *Q. suber* showed a single periderm consisting of a thick and homogeneous tissue based on suberized phellem cells (Fig. 1A). In contrast, *Q. ilex* outer bark represented the typical rhytidome displaying thin periderms consisting of few phellem cell layers (Fig. 1B). Most of the natural hybrid individuals previously identified were categorized as F1 *Q. ilex* x *Q. suber* hybrids through genetic analysis [13]. These showed a rhytidome, similar to that of *Q. ilex*, but with closer and thicker periderms and additionally, some of them presented a singular suberization of inactive phloem between periderms (Fig. 1C). In contrast, a unique hybrid individual corresponding to a backcross with *Q. suber* was identified. To our knowledge, this is the only adult backcross individual identified in the field and reported in the literature. This individual shows a unique outer bark phenotype with much thicker phellems, rather like those of cork oak (Fig. 1D), and is therefore referred to as cork-like bark, while the remaining hybrids are referred to as rhytidome-like hybrids.

**Fig. 2:**
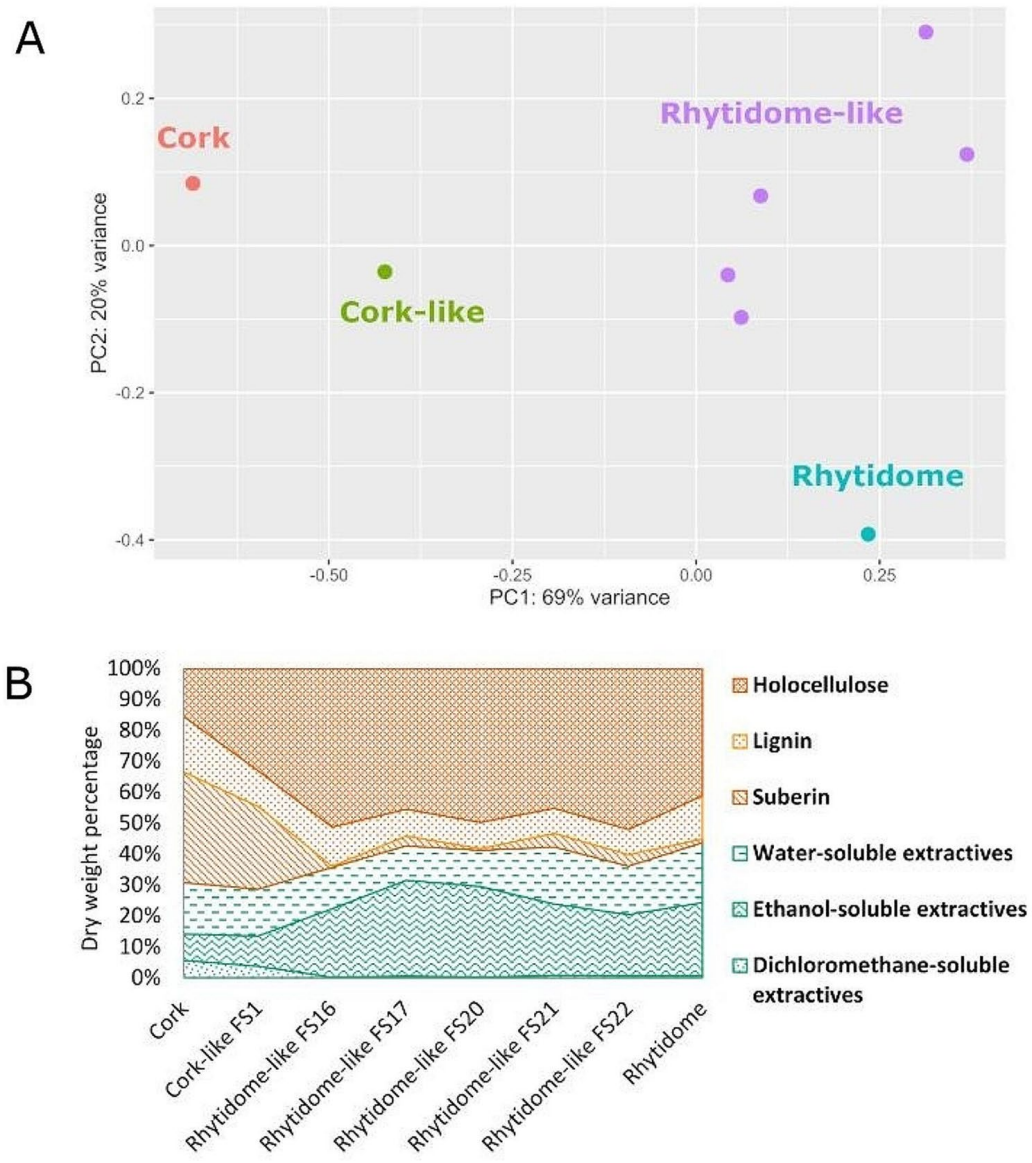
Chemical composition of the outer barks of cork oak, holm oak and their hybrids. (**A**) Principal component analysis (PCA) of the data from chemical composition analysis of the outer barks of cork oak, holm oak and the hybrids. The first principal component shows a clear separation between cork-type and rhytidome-type barks and a gradient between cork, cork-like hybrid and the rhytidome-type barks. (**B**) Dry weight % of the outer bark chemical composition of cork oak, holm oak, and a set of hybrids showing rhytidome-like bark and the hybrid showing a cork-like bark. Note the higher relative percentage of suberin and dichloromethane-soluble extractives in the cork-type barks

Consistently with these observations, the chemical analyses of the outer barks showed up different groups based on the proportion of the different components (holocellulose, lignin, suberin and water-, ethanol- and dichlorometane-soluble extractives). The cork and rhytidome-type (rhytidome-like and rhytidome) were at opposite ends of the first principal component axis of the PCA, which explained 69% of the variance (Fig. 2A). In this axis, cork-like was found between cork and rhytidome-type. A more detailed inspection of the data showed striking differences in the percentages of suberin and dichlorometane extractives across the bark samples (Fig. 2B). Specifically, the cork and cork-like outer bark samples had a ten-fold higher percentage of suberin than barks of holm oak and rhytidome-like hybrids (Table S1, Fig. 2B). Concomitantly, cork and cork-like samples presented a higher proportion in dichloromethane extractives, which contained non-polar components such as terpenes and waxes (Table S1) [14]. The abundance of both types of compounds agrees with the common fatty acyl precursors of suberin and waxes [15, 16]. Conversely, the outer bark of holm oak and the rhytidome-like hybrids contained proportionally on average 2.7 times more ethanol-soluble extractives than cork and cork-like outer barks. Interestingly, the holocellulose percentage was 3-fold higher in holm oak and all the hybrids (including the cork-like sample) than in the cork oak bark.

### Cork- and rhytidome-type barks have the most different transcriptomes

To understand the molecular processes that distinguish the outer bark composed of cork from the ones made of the rhytidome characteristic of most oak species [4], the transcriptomes of the outer bark from *Q. suber*, *Q. ilex*, and their natural hybrids (*Q. ilex* x *Q. suber*) were sequenced. Statistical results of processed data are shown in Table S2. On average, 82.61% of the reads mapped uniquely and concordantly against the cork oak genome (GCF_002906115.1_CorkOak1.0) [12] and consensus transcriptome covered 47,292 different transcripts, corresponding to 16,192 Arabidopsis (TAIR10) protein matches.

**Fig. 3:**
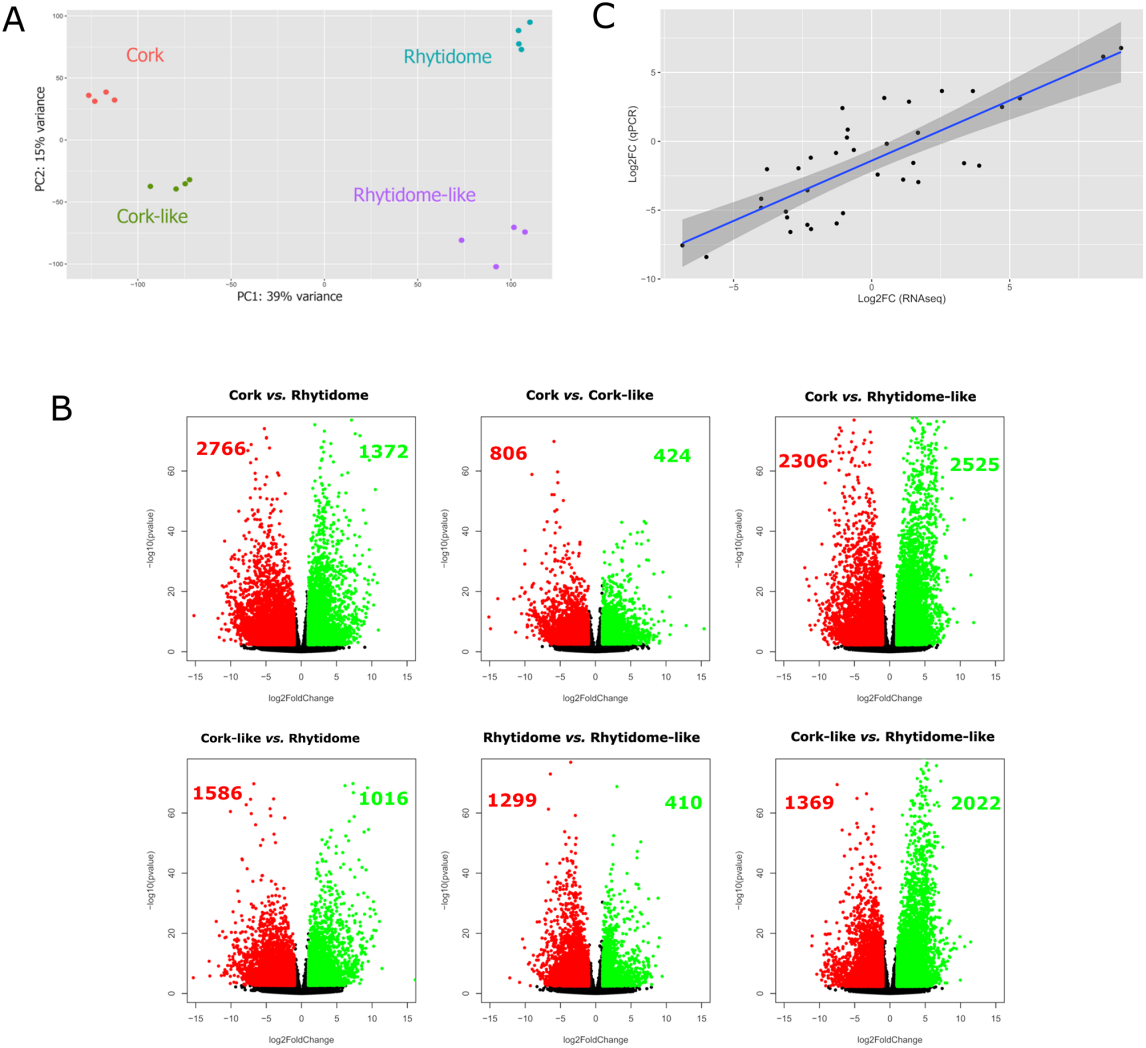
Transcriptome profile and differential expression analysis of the different outer barks. (**A**) Principal component analysis of the global transcript profile obtained from the outer barks of cork oak, holm oak and the hybrids. Similar transcriptomes within individuals of the same bark-type group together. The first principal component shows a clear separation between cork-type and rhytidome-type barks, as well as a gradient between cork, cork-like and rhytidome-type outer bark. The second component separates the rhytidome and rhytidome-like outer bark at opposite ends. (**B**) Volcano plot showing odds of differential expression (-log10 p-adjusted value) against ratio (log2 FoldChange) of different pairwise comparisons: cork/rhytidome, cork/cork-like, cork/rhytidome-like, cork-like/rhytidome, rhytidome-like/rhytidome, cork-like/rhytidome-like. Genes with –log10 greater than 2 and with log2FC absolute value greater than 1 are considered as DEGs. Green dots depict upregulated genes and red dots downregulated genes for each comparative. The number of upregulated and downregulated genes found in each comparison are shown in green and red, respectively within each graph. (**C**) Correlation graph of the mRNAs log2FC values between the RNA-seq and the qPCR analyses. The Pearson correlation coefficient (ρ) is 0.804 and the p-value < 0.001 (3.43 10^− 9^). The shaded area represents the confidence interval of the regression line

The correlation of rlog-transformed transcript profiles showed the highest similarity between biological replicates and a high similarity of cork-like outer bark with cork replicates (Fig. S1). PCA of the transcript profiles showed that the first principal component explained 39% of the total variance and distributed the cork and the rhytidome-type barks at opposite ends, and the cork-like bark was in the middle but closer to the cork one (Fig. 3A), like the chemical composition PCA. The second principal component explained 15% of the total variance in which rhytidome and rhytidome-like barks were at both ends (Fig. 3A). Next the transcriptomes of each bark type were compared to identify the differentially expressed genes (DEGs), which included those with a padj < 0.01 and a log2FC either < -1 or > 1 (Fig. 3B). Overall, 8,336 DEGs (Table S2; Fig. 3B) were found. Volcano plots showed that the comparisons with the largest number of DEGs, and thus more divergent samples, were cork/rhytidome-like (4,831 DEGs) and cork/rhytidome (4,138 DEGs) (Fig. 3B). Conversely, the comparisons presenting the lowest number of DEGs were cork/cork-like (1,230 DEGs) and rhytidome-like/rhytidome (1,709 DEGs). This is consistent with the anatomical and chemical phenotypic similarity of hybrids with their corresponding parental species.

RNA-seq data were validated by analyzing the expression of six genes in the same samples (Table S3). Log2FC of relative transcript abundance values and Log2FC of RNA-seq data presented a positive correlation (Pearson correlation coefficient of 0.804 and a p-value < 0.001 (3.43 10^− 9^)), hence confirming the RNA-seq results (Fig. 3C).

### Outer bark development: GO enrichment and the most highly expressed genes give some clues about the differential features between cork and rhytidome-type barks

**Fig. 4:**
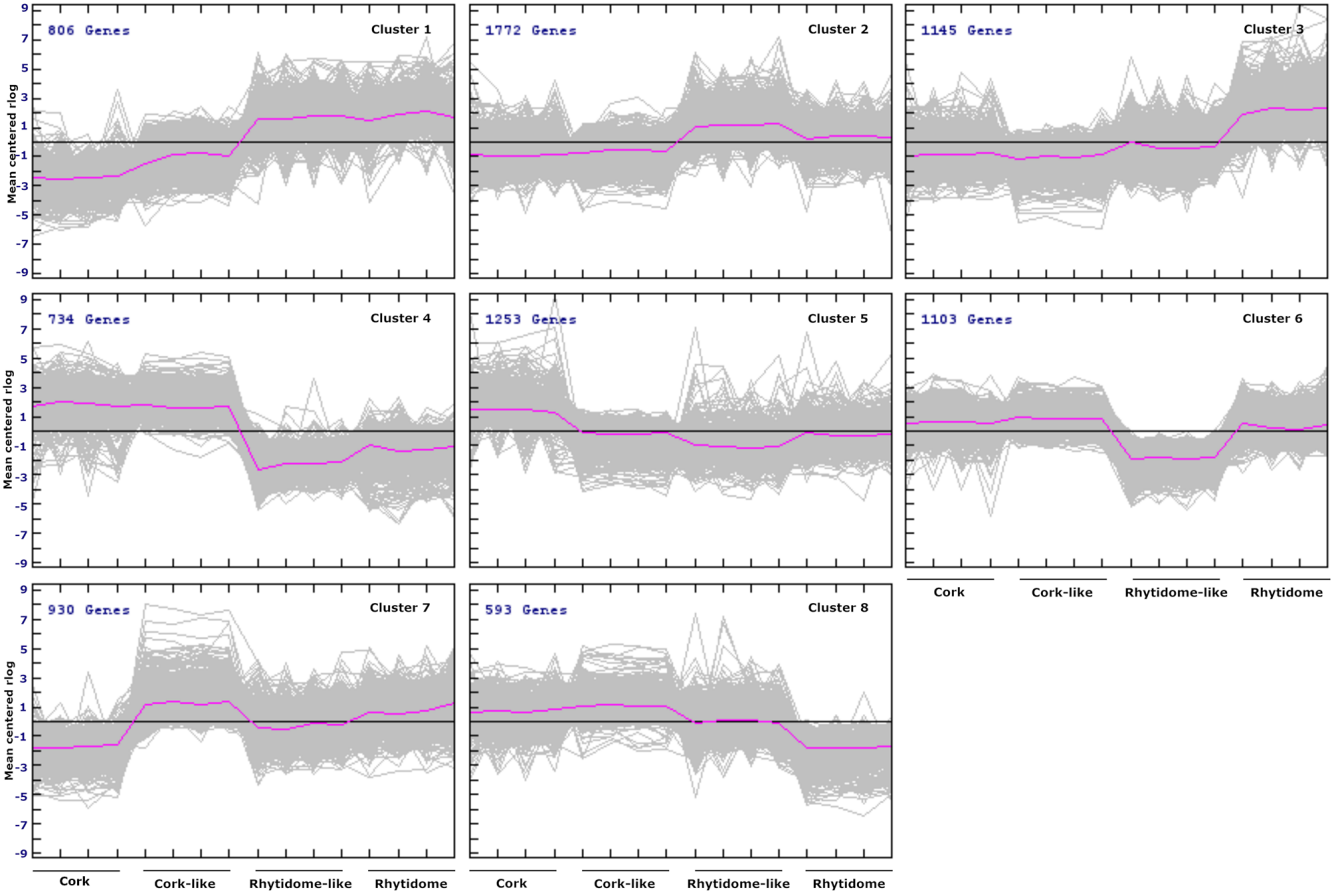
Cluster analysis of DEGs according to their expression profile in the different outer bark types. Eight clusters were obtained. Each cluster panel shows the number of genes included and the individual and averaged gene expression profile (rlog) in grey and purple lines, respectively. Clusters 1, 2 and 3 contain genes upregulated in rhytidome-type outer barks. Clusters 4, 5, 6, and 8 contain genes upregulated in cork-type barks. Cluster 7 shows particular expression peaks in cork-like and rhytidome outer barks

To identify the functional networks of proteins that distinguish bark types, the co-regulated genes were clustered and the enriched functional processes for each cluster were predicted. Based on their expression pattern, DEGs were grouped in eight clusters (Fig. 4).

Clusters with gene expression biased toward rhytidome-type barks (cluster 1, cluster 2 and cluster 3) amounted 44.7% of the total DEG. These rhytidome-type clusters were enriched in GOs related to abiotic stress, phenylpropanoid metabolism, cell death and genes classified into developmental processes (Fig. S2, Table S4).

Clusters with gene expression biased toward cork-type barks (cluster 4, cluster 5, cluster 6 and cluster 8) amounted to 44.2% of the total DEG. They were enriched in GOs related to lipid and phenylpropanoid metabolism, carbohydrates and cell wall biogenesis, oxido-reduction process, development and cell cycle (Fig. S3, Table S4).

An additional cluster (cluster 7), amounting to 11.16% of the total DEG, presented genes upregulated in cork-like and rhytidome barks and strongly downregulated in cork bark. The GO enrichment highlighted processes related to biotic and abiotic stress, cell death and senescence (Fig. S4, Table S4).

In order to identify candidate genes that regulate the formation of rhytidome-type and cork-type barks, the most expressed genes in the respective clusters among the development and cell wall related categories were selected (Table S5).

Genes related to periderm development in Arabidopsis root (*ARF6* (*auxin response factor*)), suberin monomers transport (*ABCG11* (*adenosine triphosphate binding cassete transporter type G*)), epidermal cell morphology (*Myb5* (*myeloblastosis*)), protophloem and xylem cell differentiation (*Bam3* (*Barely meristem*) and *KNAT1/BP* (*homeobox protein knotted-1-like)/ BREVIPEDICELLUS*), respectively), cell expansion reduction (*Feronia*), flowering delay (*Frigida-like* genes), repression of cell division during flower organ growth (*ARF2*), programmed cell death (*RRTF1/ERF109, redox responsive transcription factor 1/ethylene responsive factor 109*) and organ abscission (*SOBIR1, suppressor of BIR*) [17–26] stood out among the most expressed genes related to developmental process in rhytidome-type bark clusters (Table S5). Moreover, other genes highlighted as relevant for vascular patterning such as *STM* (*shoot meristemless*), *SVP* (*short vegetative phase*), *PTL* (*petal loss*), *LBD4* (*lateral organ boundaries domain*), and *LBD1* were found [23, 27–29] (Table S5). In these clusters, several genes related to suberin accumulation, with some of them even being relevant for periderm development were also identified. Specifically, an *AtMyb84* homolog, although not specifically the *QsMyb1*, two *Myb4s*, *CYP94B1 (cytochrome P450)*, *CYP94B3*, and SHR (*short root*) were detected [30–37] (Table S5). Moreover, *PER39* (*peroxidase*), which is involved in proper lignin deposition localization, was also identified [34] (Table S5).

Genes related to suberin accumulation (ASFT/FHT (*aliphatic suberin feruloyl transferase/fatty ω-hydroxyacid/fatty alcohol hydroxycinnamoyl transferase*), *CYP86B1*, *LTP1.4/LTP2* (*lipid transfer protein*)), organ growth (*MAT3* (*methionine adenosyltransferase 3*), *XTH* (*xyloglucan endotransglucosylase-hydrolase*), *glycosyl hydrolase/endo-1,4 β-D-glucanase*, *ACAT2* (*acetoacetyl CoA thiolase 2*), *HERK1* (*Hercules receptor kinase 1*), *RGP* (*reversible glycosylated protein*)), cytokinesis (*Extensin 3*), secondary wall formation of xylem cells (glycosyl hydrolase/endo-1,4 β-D-glucanase), xylem differentiation (*HB8* (*class III homeodomain-leucine zipper*)), ABA signalling pathway (*PLDα1* (*phospholipase Dα1*)) and cell wall integrity (*UGD2* (*UDP-glucose 6-dehydrogenase family protein*)) [38–53] stood out among the most highly transcribed genes related to development and cell wall biogenesis in cork-type bark (Table S5). Moreover, in all these clusters several genes related to suberin (*GPAT5* (*acyl-CoA: glycerol-3-phosphate acyltransferase*), *FAR4* (*fatty acyl-coenzyme A reductase*), *KCS2* (*3-ketoacyl-CoA synthase*), *ABCG2*, *GELP38* (*GDSL-type esterase/lipase*), *GELP51*, *GELP96*) and lignin (*PER3*, *PER72*) accumulation were also identified [34, 54–59] (Table S5). Consistent with the upregulation of those genes, several *Myb* homologs involved in the induction of suberin genes were found (*Myb9*, *Myb36*, *Myb84*, Myb93, *Myb102*, and *Myc 2* (*myelocytomatosis 2*)) [32, 33, 60–64] (Table S5). Remarkably in cluster 4, which was enriched in suberin biosynthesis, there were homologs of genes reported to repress suberin accumulation (*Myb4*, *StNAC103/AtNAC058* (*NAM/ATAF/CUC*)) [37, 65]. In addition, in this set of clusters, genes previously reported to be related to cambium activity (*AIL6* (*aintegumenta-like*, *AIL5*, *WOX4* (*Wuschel homeobox related 4*)), and phellogen activity (*WOX4*), as well as xylem differentiation (*LBD18*), and phloem differentiation (*LBD4*) were also found [26–28, 53, 66, 67] (Table S5).

## Discussion

This work takes advantage of the differences between rhytidome and cork-type bark to better understand the molecular basis of periderm formation, especially cork formation. The ontogenesis of these barks stems from an insightful difference between both types: long-living versus short-living phellogen, which turns to a homogeneous and thick periderm and a heterogeneous tissue containing thin layers of periderms, respectively. Hence, the transcriptome comparison of both types of bark is the reflection of the tissue composition and cell activity and as such can be helpful for periderm knowledge.

### Suberization is a distinctive characteristic of cork-type barks and hybrids

Analysis of the chemical composition of the outer bark regarding holocellulose, suberin, lignin and extractives content yielded results consistent with anatomical observations and transcriptomes. Specifically, the increased suberin amount in cork-type bark compared to rhytidome-type bark is consistent with the suberin proportion for these two species reported previously [68] and also with the greater number of phellem cells reported here and previously [8, 11]. In addition, the chemical composition of these outer barks also agrees with their transcriptomes, because lipid metabolism and suberin GOs are only found in cork-type bark. In agreement with this, among the cork oak bark-upregulated genes, GO enrichment of the suberin biosynthetic process was found [8].

The transcriptome comparison using outer barks showing cork or rhytidome features provided 8,336 DEGs, including those identified in hybrid individuals. Genes clearly upregulated in rhytidome-type barks were found in clusters 1, 2 and 3, while genes upregulated in cork-type barks were in clusters 4, 5, 6, and 8. Clusters 2 and 7 were specifically upregulated in hybrid individuals, with DEGs upregulated in rhytidome-like bark hybrids (cluster 2) or in cork-like bark hybrid (cluster 7).

Hybridization and introgression are well known to modify gene expression, due to the disruption of regulation pathways, mainly of trans-acting regulators, epistatic relationships or the lack of intermediate gene products acting in complex metabolic routes, for example [69–73]. This is the case of bark development, where F1 hybrids, carrying a copy of *Q. suber* genes, fail to form a long-living or persistent periderm. Maybe more interesting is the general suberization of inactive phloem, suggesting an alteration of expression patterns in this tissue, prior to its final death [11]. Genes upregulated specifically in rhytidome-type hybrids (cluster 2) may underlie this feature (Fig. S2). Considering that ABA and stress trigger suberin accumulation and the aliphatic and aromatic nature of the suberin polymer, it is tempting to speculate that cluster 2 genes belonging to GOs related to response to abiotic stress, RNA metabolism and gene expression, aromatic compound metabolism and development may be involved in the suberization of phloem cells. On the other side, the individual identified as a backcross with cork oak, the cork-like bark hybrid [13], is expected to carry, on average, two alleles coming from cork oak on half of the genes involved in bark formation. Consistently, it showed much thicker layers of phellem in its outer bark, while no suberization of inactive phloem had been detected. The clues of this thicker phellem could be in clusters 4 and 6, which corresponded to GOs related to lignin, suberin, cell wall formation, cell development and cell cycle (Fig. S3). Cluster 7 showed the most differential features between cork and cork-like bark hybrid, which GOs were biotic and abiotic response and signalling, cell death and senescence, among others (Fig. S4).

### Cell division, cell expansion and cell differentiation in the bark types

**Fig. 5:**
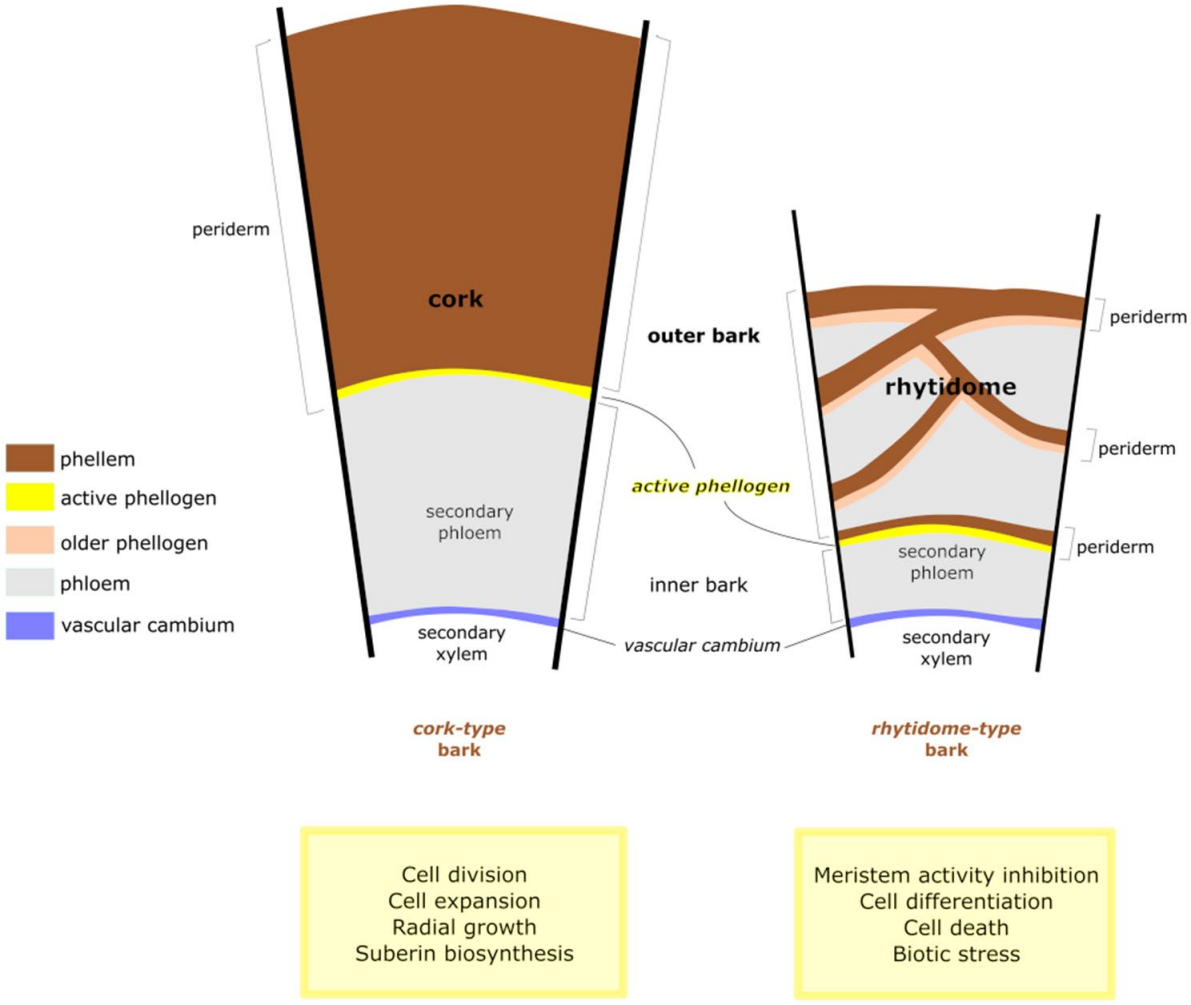
Summary of biological processes occurring during cork and rhytidome formation. This summary is based on upregulated genes and processes in cork-type and rhytidome-type outer barks from *Q. suber*, *Q. ilex* and their natural hybrids (cork-like and rhytidome-like). The outer tissue portion analysed corresponded to the inner face of the outer bark, which includes the meristematic active cells of phellogen and the alive phellem cells, and for rhytidome-type bark also included alive secondary phloem. Phellogen in *Q. suber* extends concentrically, is reactivated every growing season and forms a persistent periderm during the entire tree life called cork. In *Q. ilex*, the periderm is not persistent and is substituted for new and active phellogens formed inwardly within secondary phloem and yielding a rhytidome outer bark constituted by subsequent periderms with phloem tissue enclosed between them. The phelloderm, derived from each phellogen and located inwardly, has been omitted for simplicity; phelloderm, phellogen and phellem constitute each of the periderms depicted. Sketch inspired from Junikka [94]

Globally, the gene ontologies enriched in cork-type and rhytidome-type contained upregulated genes that displayed opposite functions referring to cell proliferation, cell expansion, and cell differentiation (Fig. 5). These contrasting gene activities align with the phenotype described for cork and rhytidome outer barks, since a major number of larger phellem cells, with higher suberin content (Fig. 2B), are produced in cork when compared with the rhytidome [8]. For rhytidome-type barks, upregulated genes related to (i) meristem activity inhibition, (ii) inhibition of cell expansion and (iii) cell differentiation were identified. For example, regarding the most expressed and upregulated genes in rhytidome-type barks, genes that inhibit cell division (i) such as *ARF2*, *BAM3*, *SVP*, *PTL* and *LBD1* were detected. *ARF2* is a repressor of cell division and flower organ growth [18]. *BAM3*, also could inhibit cell division because the null mutation in *BAM3* suppress the postembryonic root meristem growth defect of *BRX* (*brevis radix*) mutant [21]. SVP and PTL inhibit vascular cambium activity [28] and PtLBD1 suppresses the vascular cambium cell identity and promotes phloem differentiation [27]. In relation to cell expansion inhibition (ii), *FERONIA* was identified. Feronia reduces cell expansion by binding to RALF (rapid alkalinization factor) and increasing the apoplastic pH [22], as well as promoting crosslinking between cell wall pectins by pectin de-esterification [74]. Concerning cell differentiation (iii), several positive regulators of triggering cell differentiation over meristematic cell state were upregulated such as *LBD1* (mentioned above), *BAM3*, *ARF6*, *KNAT1/BP*, *STM*, and *LBD4*. *BAM3* was proposed to participate in the differentiation of protophloem [21]. *ARF6*, expressed in all stages of root periderm development in Arabidopsis [26], induces vascular patterning and epidermal cell differentiation through negative regulation of class 1 *KNOX* genes [75]. About these *KNOX* genes, *KNAT1/BP* was identified, that, despite promoting vascular cambial activity [28] and increasing the number of periderm cell layers in the root [26], it has an opposite role in the hypocotyl by promoting xylem differentiation together with *STM*, another class I *KNOX* gene [23], which was also upregulated in rhytidome-type samples. *LBD4* was considered a major node in the network of vascular development [28] related to phloem recovery defects, possibly acting as a boundary regulator or as an amplifier of divisions on the phloem side of the procambium [29]. Conversely, regarding cork-type barks, genes (i) promoting cell division (*AIL6*, *HB8*, *AIL5*, *RGP*, *EXT3*, *cyclins*, and *cyclin-dependent kinase*) and meristem maintenance (*AIL6*, *glycosyl hydrolase*, *WOX4*, *HB8*) and, (ii) some genes involved in cell expansion (*XTHs*, *ACAT2*, *ERK1*, and *expansins*) and (iii) radial growth (*LBD4* and *LBD18*) were identified, supporting the superior cell size and cell production of phellem layers in cork oak. Regarding meristem activity (i), AIL6, together with ANT and AIL7, is required for meristem maintenance, by promoting cell division and repressing cell differentiation in shoot apical meristem [66]. The glycosyl hydrolase is a membrane-bound endo-1,4 β-D-glucanase involved in cellulose synthesis necessary for maintaining meristematic pattern, organ growth in shoot and root and hormone response [47], that can regulate cortical microtubule organization [76]. As concerns to WOX4, it has been shown that it promotes phellogen activity in root periderm [26] and HB8 inhibits cell division and promotes cellular quiescence in the vascular cambium stem-cell organizer, located at the xylem side of the vascular cambium, but able to maintain xylem and phloem identity at both sides [53]. These results allow us to speculate that HB8 would induce a similar dynamic organizer within the phellogen stem cell population, which would also accumulate WOX4, as reported for vascular cambium [53]. Cork-type barks also showed upregulation of genes involved in cell division and/or cell plate formation such as *RGP* and *EXT3* [39, 40]. As regards genes inducing cell expansion (ii), XTH is able to modify xyloglucans chains, which turnover is required during cell and organ elongation [41, 77]. ACAT2 catalyses the formation of mevalonate-derived isoprenoids with consequences on the proper growth of vegetative tissues and a special effect on cell number in the xylem and phloem [50]. HERK1 is a receptor-like kinase (RLKs) shown to be involved in cell expansion by regulating xyloglucan endotransglucosylase/hydrolases and expansins [42]. According to this function, several x*yloglucan endotransglucosylase/hydrolase* and *expansins* were also found upregulated in cork-type barks and specifically in the same cluster. Finally, several LOB domain-containing proteins involved in radial growth [28] were upregulated in cork-type barks. It was shown that one of them was expressed in the secondary phloem (*LBD4*) and the other in secondary xylem (*LBD18*) and it was suggested that *LBD4* was involved in recruiting cells into the phloem lineage while defining the phloem-procambium boundary [27, 29].

### Cell abscission-related processes correlate with rhytidome-type bark features

One of the most striking differences between rhytidome- and cork-type barks is the shedding of outer bark layers from rhytidome and the ability to keep one unique persistent periderm within yearly produced phellem cells. It is highly remarkable *SOBIR1*, upregulated in rhytidome, which was recently suggested to contribute to organ abscission by transducing the signal downstream of SERK proteins [25]. Organ abscission is a precisely controlled process that gives rise to cell wall loosening and degradation of cell wall components, being pectin-rich middle lamella the major physical mediator of cell adhesion and separation [78]. Besides, organ abscission is induced by jasmonic acid, which overlaps with defence processes [79], and lignin deposition also takes place to the abscised region limit to restrict cell wall hydrolyzing enzymes [80]. It is worth mentioning that cluster 3, induced in rhytidome-type barks and with a peak in rhytidome, in which *SOBIR1* is found, is enriched in biotic stimulus, lignin, jasmonic acid, and cell wall biogenesis. The cell wall-related genes identified in this study can be insightful and it is tempting to speculate that cell abscission is an active process leading to rhytidome-type bark.

## Conclusions

The main goal of the present study is to provide insight into the molecular mechanisms driving the development of different types of outer bark in woody species, namely the most common rhytidome (characterized by anastomosed thin periderms, encompassing sectors of lignified dead phloem) and the unique, thick phellem typical of *Q. suber* and few other species which present a single long-lived or persistent phellogen. For this purpose, chemical, anatomical and transcriptomic approaches have been performed in *Q. ilex* (rhytidome), *Q. suber* (thick cork) and hybrid samples. Analysis of the chemical composition of these bark types is consistent with the anatomical observations, with *Q. suber* yielding a larger suberin amount, while hybrid samples show different intermediate situations. The inclusion of hybrids has allowed us to highlight 8,336 DEGs. It is shown that cork-type barks are enriched in GOs related to lipid metabolism and cell cycle while rhytidome-type barks are mostly enriched in GOs related to abiotic, biotic stress and cell death. Focusing on cell wall biogenesis and development, genes promoting meristem activity and cell expansion are upregulated in cork-type barks, while rhytidome-type barks show higher expression of genes inhibiting cell division and expansion and promoting cell differentiation. Further research is needed to disentangle the regulatory pathways of the candidate genes identified in this work, as well as their additive and non-additive effects on bark development.

## Methods

### Outer bark harvesting

Outer barks of tree trunks from four adult cork oaks (*Quercus suber* L.) and four holm oaks (*Quercus ilex spp rotundifolia*) were harvested. Six and five *Q. ilex* x *Q. suber* hybrids were sampled for the chemical and transcriptomic analyses, respectively. The cork oak trees had not been previously decorticated, so virgin cork was used for the analyses. Trees were naturally grown in a mixed holm oak-cork oak forest in Fregenal de la Sierra (Extremadura, Spain). These hybrids were previously identified according to morphological features and molecular markers and a detailed anatomy was reported recently [11, 13]. The samples were obtained when the phellogen activity was high enough to allow the outer bark detachment from the inner bark. For each cork oak, holm oak and rhytidome-like groups, four south-oriented bark samples each from a different tree were sequenced. For the cork-like hybrid unique individual, four bark samples extracted from the north, south, west, and east orientations were sequenced. Outer bark was harvested at breast height, and it was manually removed from the trunk using a hammer and a chisel. The material was collected from the inner face of the outer barks scratching with a chisel, immediately frozen in liquid nitrogen, and kept at -80 ºC for further use. Anatomical observations were performed as detailed in de Burgos et al. [11].

### Chemical analysis of the outer barks

Chemical analyses were performed in one representative sample of cork and rhytidome, five samples of rhytidome-like bark hybrids and one sample from the cork-like bark hybrid. The summative chemical analyses included the determination of ash, extractives, suberin, Klason lignin, and holocellulose. The ash content was determined by incinerating 2 g of cork at 525ºC for 1 h with a muffle furnace (Faenza, Italy). Extractives were determined by successive Soxhlet extraction with dichloromethane (6 h), ethanol (8 h) and hot water (20 h). After each extraction, the cork residue was air-dried and kept for subsequent analysis and the extracted solution was evaporated to obtain the solid residue, which was weighed. The suberin content was determined in extractive free material by alkaline methanolysis for its depolymerisation using a Soxhlet in reflux mode for 3 h. Then, the extracted liquid was acidified with 2 M H_2_SO_4_ to pH 6 and evaporated to dryness in a rotating evaporator (Aircontrol, Spain). This residue was suspended in 100 ml H_2_0 and extracted with 100 ml CHCl_3_ three times. The combined extracts were dried over Na_2_SO_4_, filtered, evaporated, and determined gravimetrically as suberin. On the other hand, the desuberized solid material was used for Klason lignin determination by hydrolysis with 72% H_2_SO_4_ [81]. Holocellulose fraction was isolated from desuberized fraction by delignification using the acid chloride method [82]. All measurements were reported as a percentage of the original sample. Principal components analysis (PCA) was performed to plot the variation of outer bark chemical composition using the log-transformed data of the percentage of each fraction (variables) in the eight samples.

### Total RNA extraction and purification

Total RNA was extracted from outer barks using a modified method described previously [83, 84]. Two grams of tissue were grounded in liquid nitrogen using a mortar and pestle and rapidly mixed with 15 ml of preheated (65 ºC) CTAB extraction buffer (2% CTAB, 4% PVP-40, 300 mM Tris-HCl pH 8.0, 25 mM EDTA, 2 M NaCl, and 3.3% 2-mercaptoethanol) using a vortex. After a 10-min incubation at 65 °C, the sample was extracted twice with one volume (V) of chloroform: isoamyl alcohol 24:1 (v: v), followed by centrifugation at 15,000 *g* for 20 min. The aqueous fraction was precipitated using 1 volume of isopropanol and 0.1 volume of NaOAc 3 M (pH 5.2) and incubated for 3 h at -80 °C or overnight at -20 °C. The precipitate was collected by centrifugation at 15,000 *g* for 30 min, resuspended in 700 µl of preheated (65 °C) SSTE buffer (1 M NaCl, 0.5% SDS, 10 mM Tris-HCl pH 8 and 1mM EDTA), treated twice with the same volume of chloroform: isoamyl alcohol (24:1 (v: v)) and centrifuged 10 min at 21,000 *g*. The supernatant was precipitated overnight with 2 volumes of ethanol 100% at -80 °C and collected by centrifugation at 21,000 g for 30 min at 4 ºC. After two washes with 70% ethanol, the nucleic acid pellet was resuspended in 50 µl of RNase-free water. RNeasy Power Plant Kit (Qiagen) and DNAse I on-column digestion were used to remove polyphenols and genomic DNA, respectively. Briefly, 500 µl of MBL (99: 1 MBL: β-mercaptoethanol) was added to 50 µl of each total RNA sample together with 50 µl of PSS and 200 µl of IRS. The total RNA yield was measured with a Nanodrop and the RNA integrity values (RIN) were obtained with a Bioanalyzer 2100 (Pico RNA 6000 Kit, Agilent). The values obtained for each of the samples are shown in Table S2.

### Analysis of RNA-seq high-throughput mRNA sequencing data

Outer bark cDNA libraries were obtained using the MGIEasy RNA Library Prep Kit V3.1 and 3 µg of each sample (RIN value > 8). Sequencing was performed by the BGISEQ500 (paired-end reads of 100 bp) at BGI Genomics (Hong Kong). In total, 16 samples were sequenced. A minimum of 100 M reads was obtained for each library. The quality of raw reads was assessed with FASTQC software [85] and removal of the first low-quality 12 bp was performed with Trimmomatic [86]. The reads were mapped with GSNAP [87] against the *Q. suber* genome as a reference (GCF_002906115.1_CorkOak1.0_genomic.fna) [16], and the unique concordantly mapped reads were kept for library construction. Willing to work with unique gene identifiers, different isoforms were collapsed using the genome positions and total counts were estimated by HTSeq-count [88]. PCA analysis of transcript profiling was conducted using the plotPCA function of DESeq2 [89] on the count data after variance stabilizing transformation. The results were customized and displayed in bivariate diagrams showing the main factors displayed by ggplot2 [90].

The count matrix was generated using the 16 libraries and allowed to identify differentially expressed genes (DEGs) using the DESeq2 package [89]. DEGs were obtained from raw read counts by pairwise comparison: cork (cork oak bark) vs. rhytidome (holm oak bark), cork vs. cork-like bark (hybrid bark similar to cork), cork vs. rhytidome-like bark (hybrid bark similar to rhytidome), cork-like vs. rhytidome, rhytidome-like bark vs. rhytidome and cork-like vs. rhytidome-like. Genes with an adjusted p-value smaller than 0.01 and log2FC ≤ -1 and ≥ 1 were considered as DEGs. DEGs were clustered using the MeV program [91]. The count data (rlog) was mean centered by gene and analyzed by k-means and Euclidean distance. To obtain the *Arabidopsis thaliana* homologs, Blastp and the TAIR10 library from Ensembl were used, with the options num_alignments 1 and e-value 1e^− 08^. AgriGO V2.0 [92] was used for gene ontology enrichment for the best Arabidopsis homologs (FDR ≤ 0.05). The GO terms were manually collapsed based on the analogous description and the set of genes they contained.

### Real-time quantitative PCR

The analysis was performed in all biological replicates using primers for six genes (Table S3). First-strand cDNA was synthesized from 200 ng DNase digested RNA using RevertAid First Strand cDNA Synthesis Kit (Thermofisher). The synthesis of cDNA was performed using oligodT primer and following manufacturer’s instructions. The program for the cDNA synthesis was as follows: 16 °C for 30 min; 60 cycles of 30 °C for 30 s, 42 °C for 30 s and 50 °C for 60 s; 85 °C for 5 min. Real-time PCR analysis was performed using a LightCycler® 96 Real-Time PCR System (Roche). Primers were designed for each gene with Primer3-0.4.0 software (http://bioinfo.ut.ee/primer3-0.4.0/). Each RT-qPCR reaction (10 µl) contained 5 µl of SYBR Green Select Master Mix (Roche), 300 nM of the corresponding forward and reverse primers, and 2.5 µl of a 25-fold diluted cDNA. The conditions of the thermal cycle were the following: 95 °C for 10 min; 40 cycles of 95 °C for 10 s and 60 °C for 60 s. A final dissociation step was included to confirm a single amplicon. For each primer pair, standard curves with a five-fold dilutions series of a cDNA mix corresponding to equal amounts of all biological replicates of cork bark, rhytidome bark, cork-like bark, and rhytidome-like bark (1/10, 1/25, 1/50, 1/100, and 1/250) were used to determine amplification efficiency of each gene (E = 10 ^(-1/slope)^). The mRNA abundances for each gene were calculated as relative transcript abundance = (E_target_)^ΔCt target (control-sample)^ / (E_reference_)^ΔCt reference (control-sample)^ [93]. The calibrator or control sample consisted of equal amounts of cDNA of all biological replicates. The housekeeping gene used to normalize the results was tubulin [94]. DNA contamination of the samples was ruled out using non-retrotranscriptase controls, and to confirm no presence of environmental contamination, non-template controls were included in each experiment. Three technical replicates were used for every four biological replicates.

### Electronic supplementary material

Below is the link to the electronic supplementary material.

Supplementary Material 1

Supplementary Material 2

Supplementary Material 3

Supplementary Material 4

## Data Availability

All data supporting the findings of this study are available within the paper, within its supplementary materials published online and in the Gene Expression Omnibus repository from NCBI under accession code GSE227020. The material used in this study will be shared on reasonable request to the corresponding author.

## Abbreviations

CTAB: Cetyltrimethylammonium bromide
DEGs: Differentially expressed genes
EDTA: Ethylenediaminetetraacetic acid
FC: Fold change
PCA: Principal component analysis
FDR: False discovery rate
GO: Gene ontology
PVP: Polyvinylpyrrolidone
