# Supplementary material for: Rhytidome- and cork-type barks of holm oak, cork oak and their hybrids highlight processes leading to cork formation
Source: BMC Plant Biol. 2024 Jun 3;24:488. doi: 10.1186/s12870-024-05192-4 (PMC11145776; doi:10.1186/s12870-024-05192-4)
Supplement: Supplementary file 4 — Supplementary Material 4 [file 12870_2024_5192_MOESM4_ESM.pdf]

SUPPLEMENTARY INFORMATION

Rhytidome- and cork- type barks of holm oak, cork oak and their hybrids highlight processes leading to cork formation

Iker Armendariz, Unai López de Heredia, Marçal Soler, Adrià Puigdemont, Maria Mercè Ruiz-Sans, Patricia Jové, Álvaro Soto, Olga Serra, Mercè Figueras.

Table S1. Chemical composition of outer bark (%) of cork oak (cork, *Quercus suber*), holm oak (rhytidome, *Quercus ilex*) and the *Q. ilex* x *Q. suber* hybrids. There are five hybrids showing a rhytidome-like bark (FS16 to FS22) and one showing a cork-like bark (FS1).

**Samples**	***Ash content***	***Total extractives content***			***Suberin***	***Lignin***	***Holocellulose***
		Dichloromethane	***Ethanol***	***Water***			
Cork	0.8	5.6	8.4	16.5	35.4	17.8	15.4
Cork-like FS1	0.9	3.7	9.7	15	26.6	11.4	32.7
Rhytidome-like FS16	1.0	0.3	21.8	13.3	0.4	12.5	50.8
Rhytidome-like FS17	0.9	0.6	30.7	11.0	3.2	8.6	45.1
Rhytidome-like FS20	1.0	0.2	29.0	11.6	0.6	8.3	49.3
Rhytidome-like FS21	1.0	0.8	22.8	18.2	4.4	8.0	44.7
Rhytidome-like FS22	1.0	0.7	19.6	15.5	3.7	8.0	51.6
Rhytidome	0.9	0.7	23.4	19.3	1.2	13.8	40.7

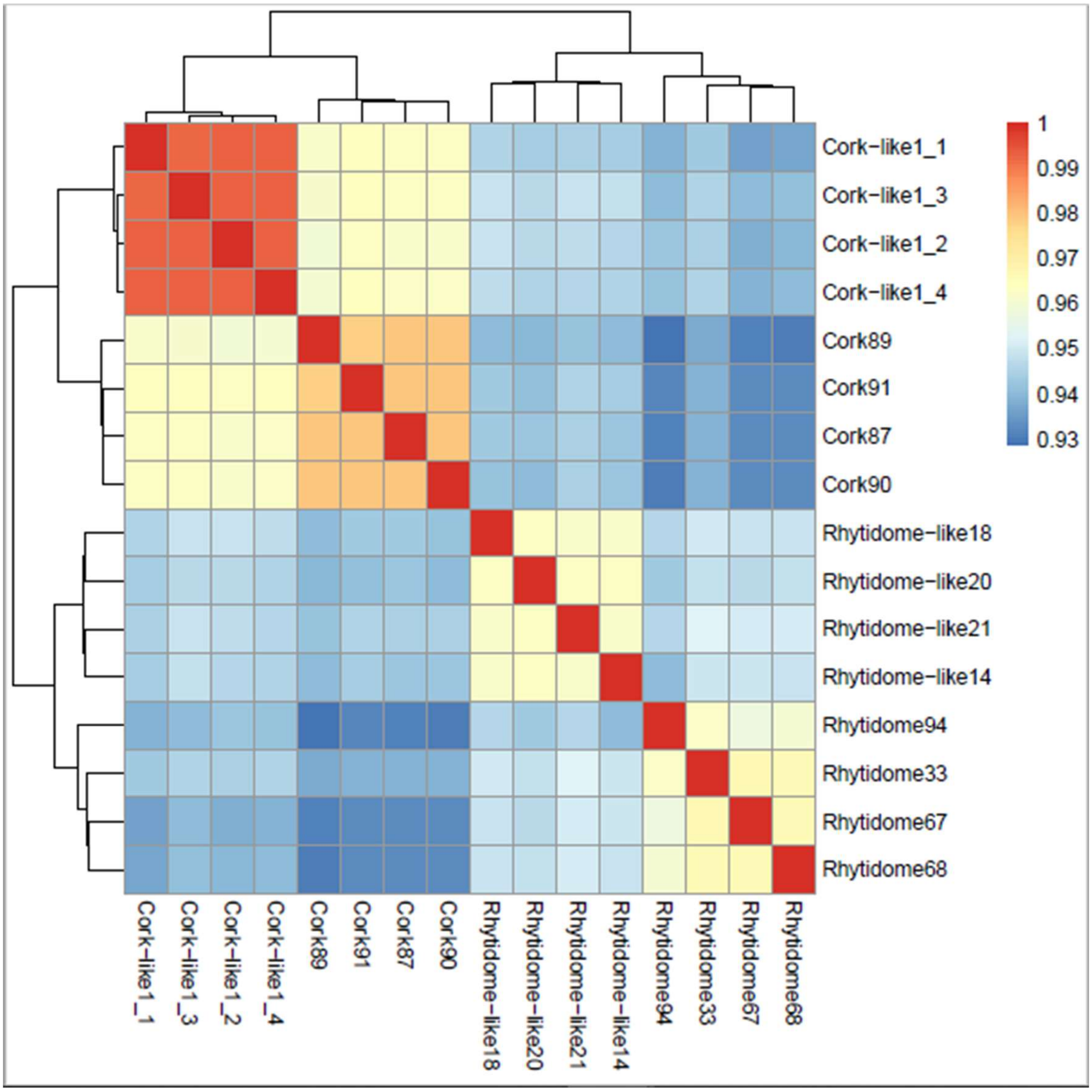

Fig. S1. Correlation map of different outer bark transcriptome profiles. Rhytidome and cork correspond to the outer barks from holm oak and cork oak, and rhytidome-like and cork-like to the outer barks of hybrids. Numbers correspond to the tree identification number.

Table S3. Primer sequences used for RNA-seq validation by Real Time PCR.

Gene Name	*Quercus suber* identifier	Best Arabidopsis homolog (BlastX)	Primer sequences (from 5'- to 3'-end): forward (Frw) and reverse (Rev) primers
RBOHD (respiratory burst oxidase homologue D)	LOC112002845	AT5G47910	Frw: AACTGGACAACAGGGGAGTG<br>Rev: CTTGGCATGATTGAGGGACT
SNF1-RPK (SNF1-related protein kinase regulatory subunit beta-2)	LOC111986784	AT5G21170	Frw: AGAAGGTGTGGAAGGTGGTG<br>Rev: GACCCATCAAGTCAGGAGGA
ZFP ZAT10-like (zinc finger protein ZAT10-like)	LOC112003415	AT1G27730	Frw: TCAGCCAATACCAACACCAA<br>Rev: ACCGCCTTCAGAGAAAGTGA
aspartyl protease	LOC111989591	AT2G17760	Frw: GTGCTTTGGGATTTTTGCAT<br>Rev: GTAAGGCCTTTACGCAACCA
Frigida-like	LOC111986594	AT5G48385	Frw: GTGCTCAGGTGCATTGAAGA<br>Rev: CTCTTTGGTTGAGGCTTTGC
GRP DC7.1-like (glycine-rich protein)	LOC112034238	-	Frw: GCAGTGAATGAGGACAAGCA<br>Rev: AAGTTCCTCTCCAGCAGCAC
tubulin beta-2 chain-like	LOC112004091	AT2G29550	Frw: AAGAACATGATGTGCGCTGCT<br>Rev: TCCACCTCCTTGGTGCTCA

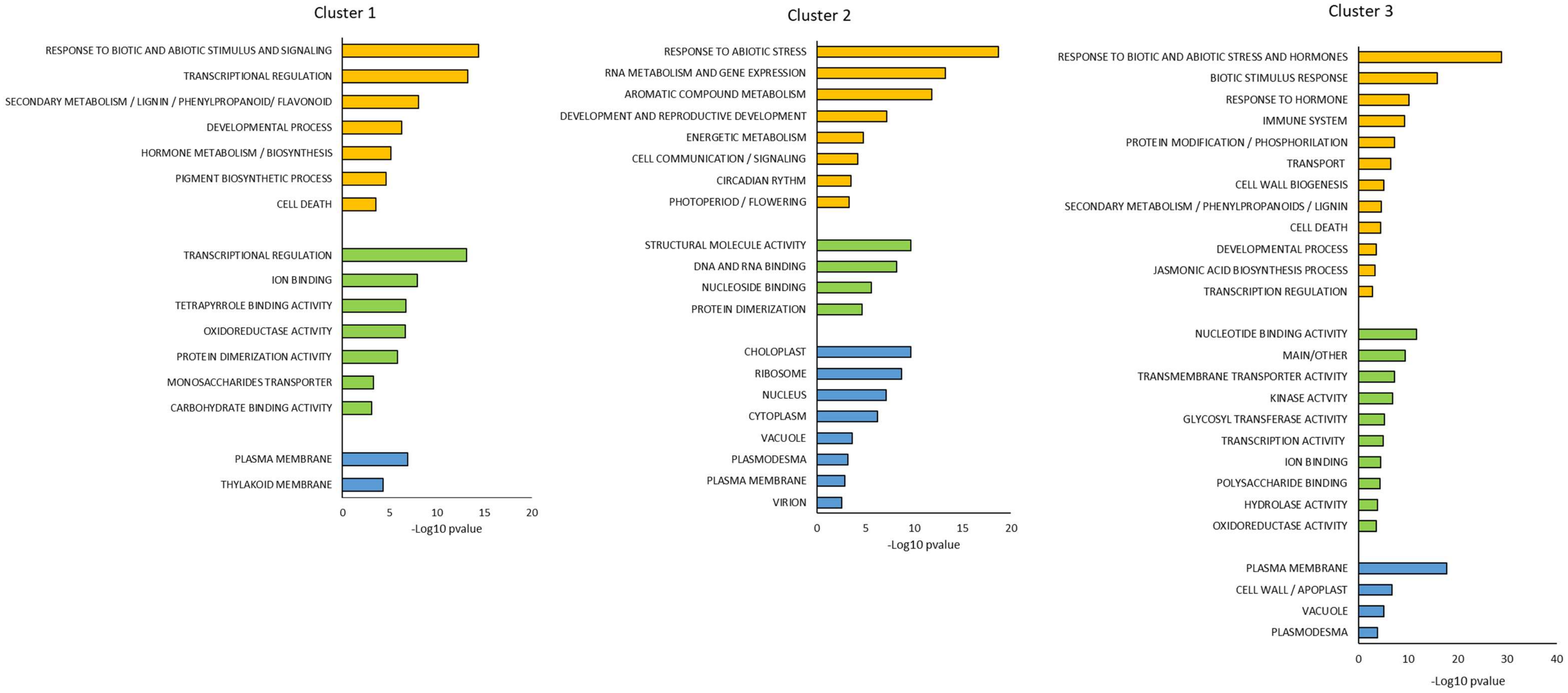

Fig. S2. Gene ontology enrichment for genes upregulated in rhytidome-type barks (clusters 1, 2 and 3). Bars represent the log10 p-value for each GO term. The GO terms were manually compared and those showing the analogous description and same set of genes were grouped, the log10 p-value corresponds to the broader GO term (including the maximum number of genes). The terms are biological process (yellow), molecular function (green), and cell component (blue).

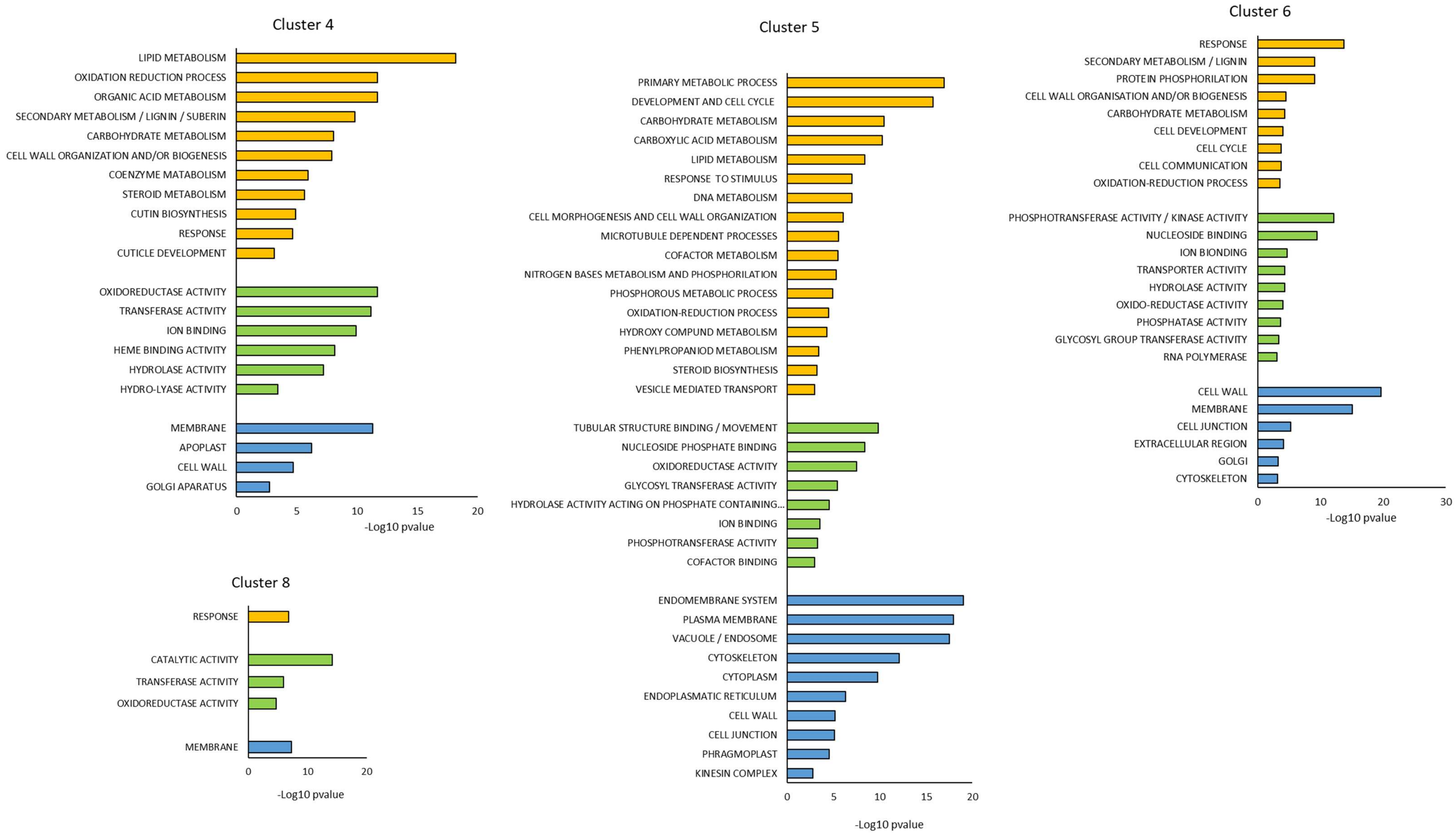

Fig. S3. Gene ontology enrichment for genes upregulated in cork-type barks (clusters 4, 5, 6 and 8). Bars represent the log10 p-value for each GO term. The GO terms were manually compared and those showing the analogous description and same set of genes were grouped, the log10 p-value corresponds to the broader GO term. The terms are biological process (yellow), molecular function (green), and cell component (blue)

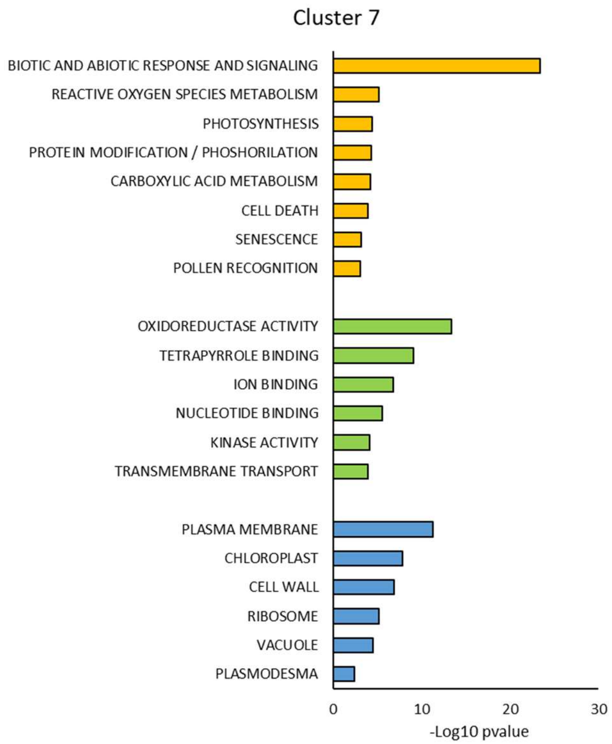

Fig. S4. Gene ontology enrichment for genes found in cluster 7. In this cluster genes are upregulated in cork-like and rhytidome bark and downregulated in cork bark. Bars represent the log10 p-value for each GO term. The GO terms were manually compared and those showing the analogous description and same set of genes were grouped, the log10 p-value corresponds to the broader GO term. The terms are biological process (yellow), molecular function (green), and cell component (blue).
